# Supplementary material for: Surface Persistence of Trace Level Deposits of Highly Energetic Materials
Source: Molecules. 2019 Sep 26;24(19):3494. doi: 10.3390/molecules24193494 (PMC6804148; doi:10.3390/molecules24193494)
Supplement: Supplementary file 1 [file molecules-24-03494-s001.pdf]

Surface persistence of trace level deposits of highly energetic materials

Leonardo C. Pacheco-Londoño[1,2*], José L. Ruiz-Caballero[1,3,4], Michael L. Ramírez-Cedeño[1], Ricardo Infante-Castillo[4], Nataly J Gálan-Freyle[1,2], and Samuel P. Hernández-Rivera[1]

1. Micro images of the morphology of HEM residues on SS substrates surfaces.

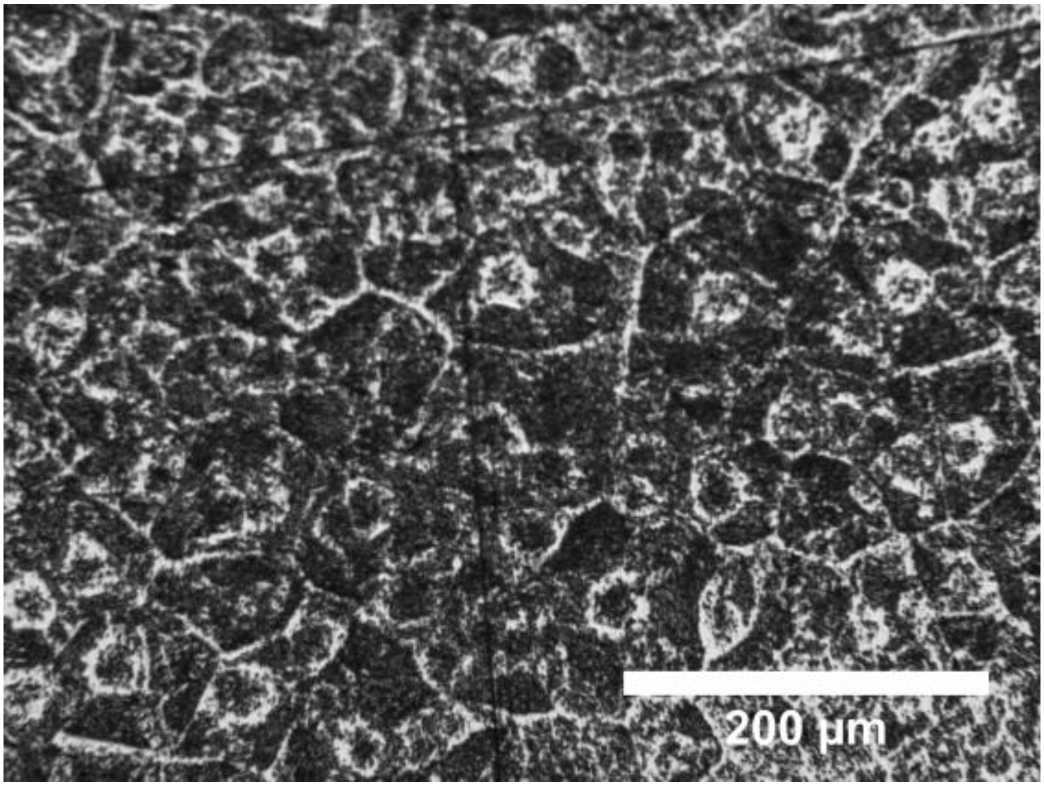

SM Fig. 1. 10x magnification micro image of morphology of residues of TATP on a SS substrate surface.

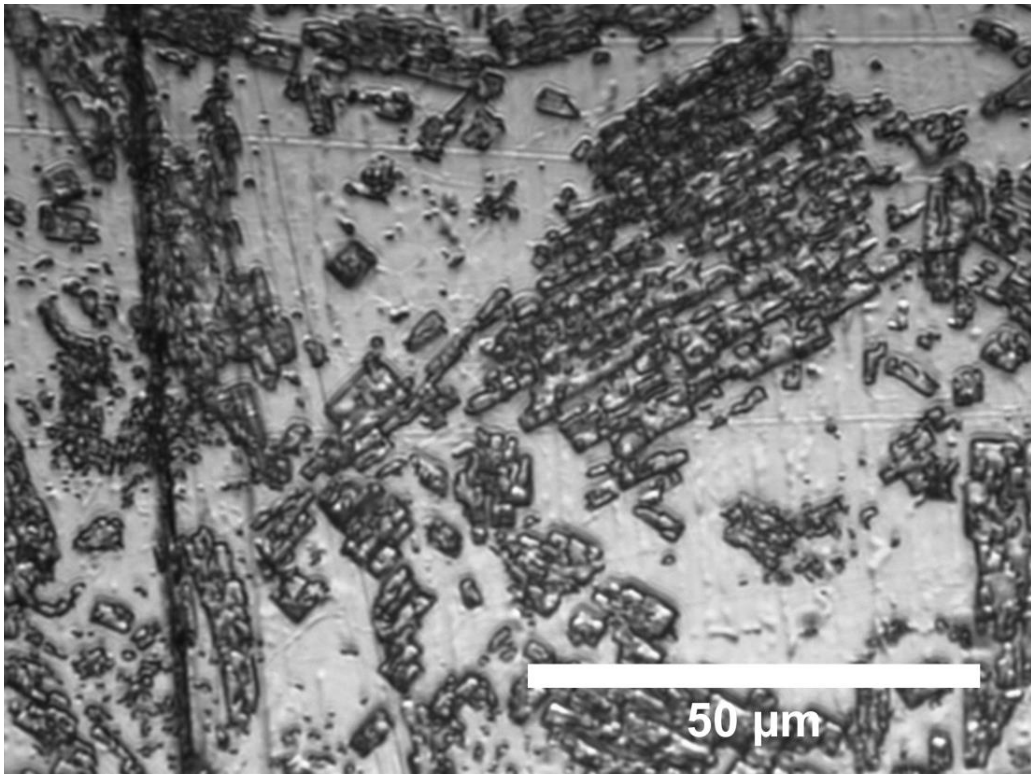

SM Fig. 2. 50x magnification micro image morphology TATP residues on a SS substrate surface.

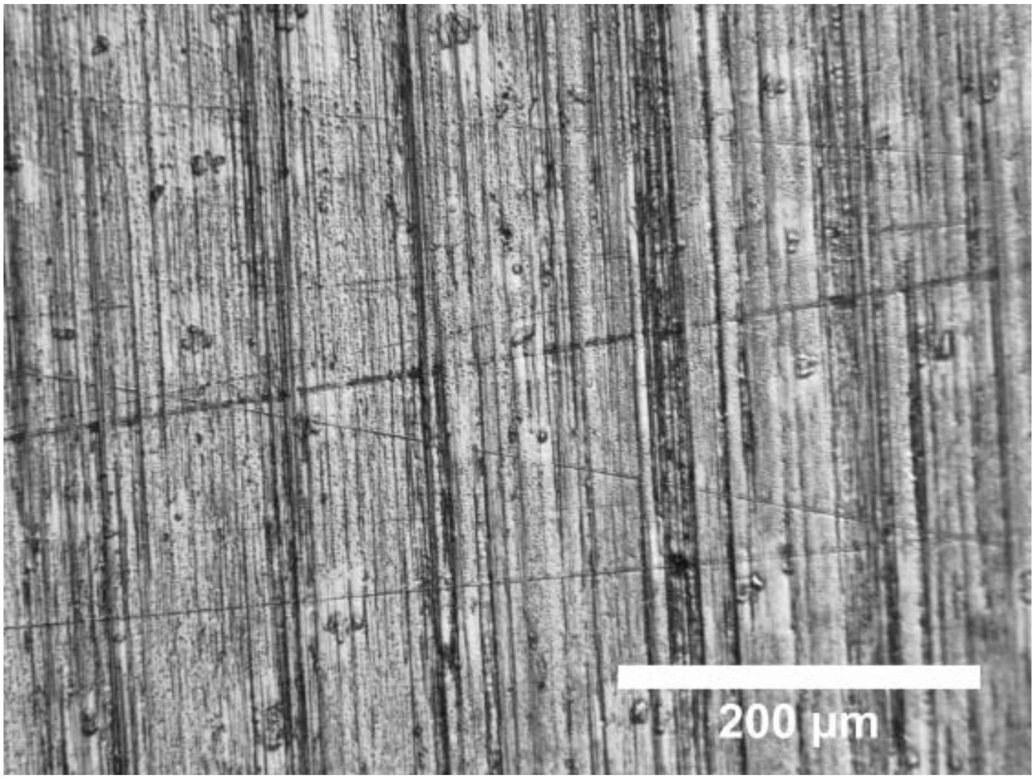

SM Fig. 3. 10x magnification micro image of morphology of DNT residues on a SS substrate surface.

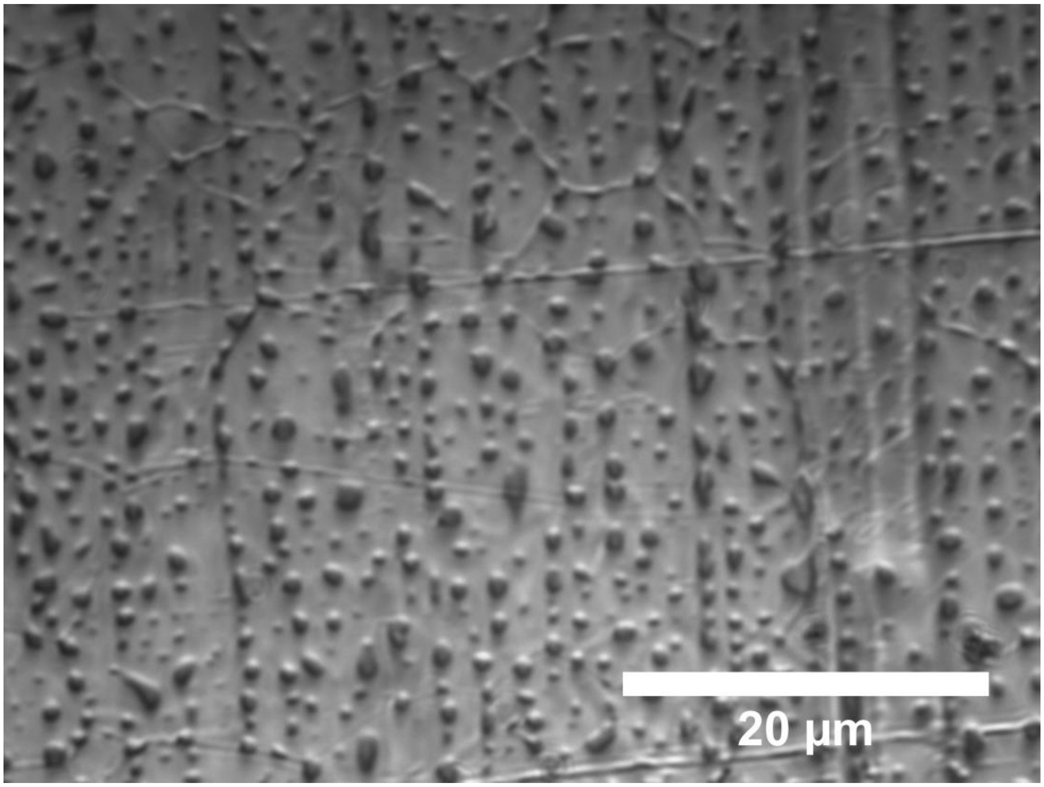

SM Fig. 4. 100x magnification micro image of morphology of DNT residues on a SS substrate surface.

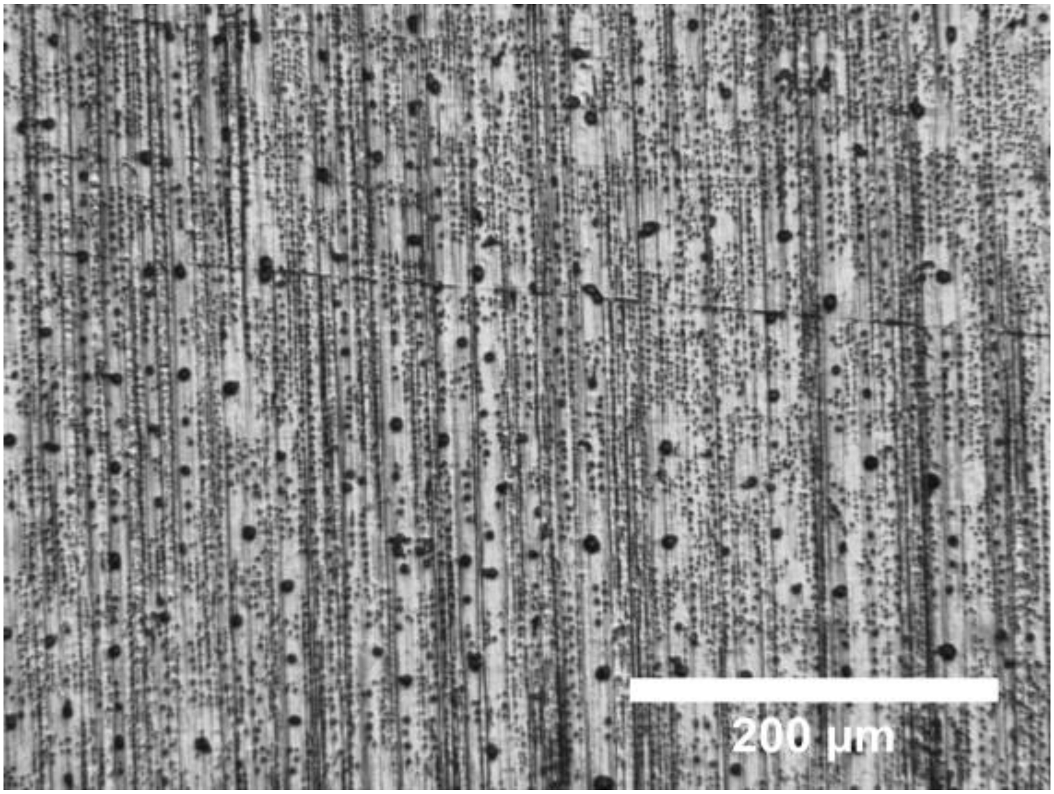

SM Fig. 5. 10x magnification micro image of morphology of TNT residues on a SS substrate surface.

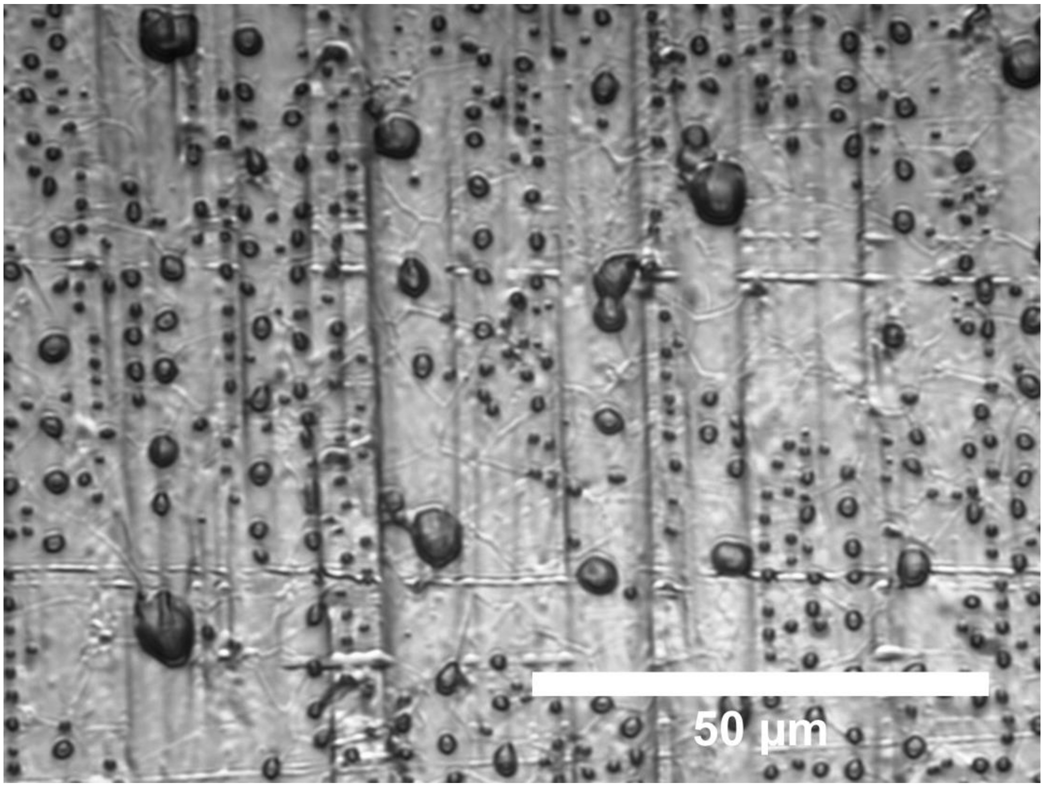

SM Fig. 6. 50x magnification micro image of morphology of TNT residues on a SS substrate surface.

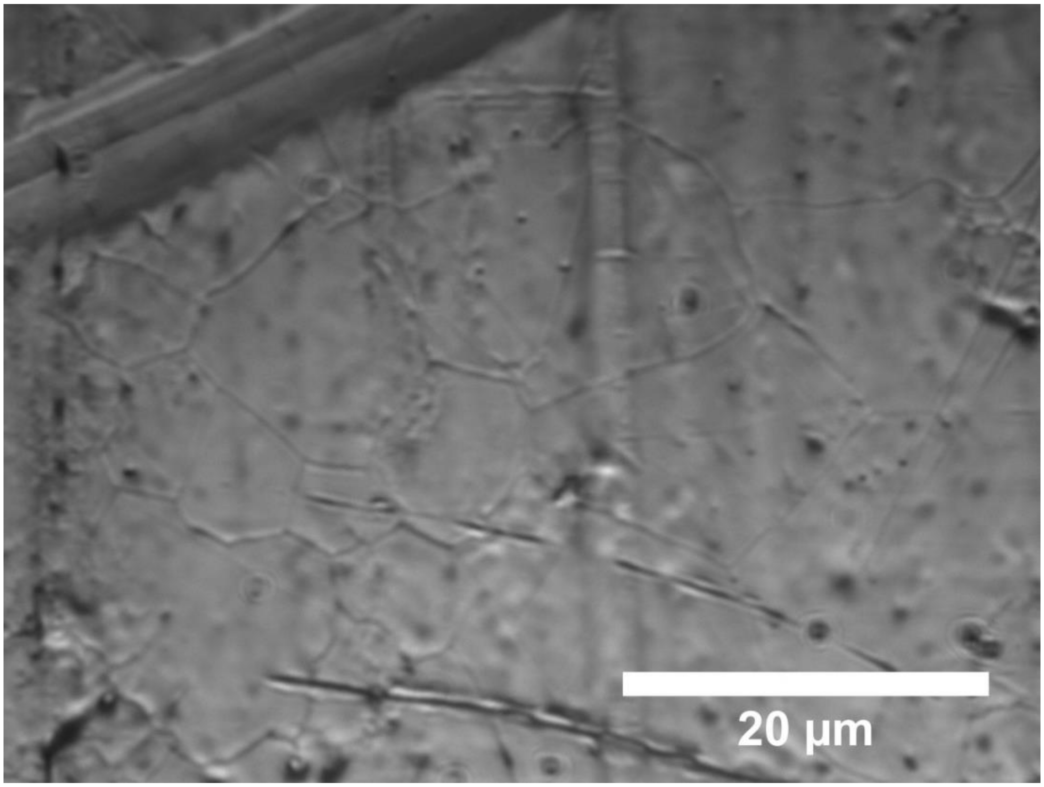

SM Fig. 7. 10x magnification micro image of morphology of RDX residues on a SS substrate surface.

2. Sublimation constants for explosive from GAP measurements.

SM TABLE I. Sublimation constants for explosive from GAP measurements

TATP		2,4-DNT		TNT		RDX	
T (°C)	k (s^{-1})	T (°C)	k (s^{-1})	T (°C)	k (s^{-1})	T (°C)	k (s^{-1})
14	0.002 ± 0.001	23	0.0007 ± 0.0003	22	(1.35 ± 0.01)E-5	22	(2.0 ± 0.2)E-8
15	0.002 ± 0.002	26	0.0010 ± 0.0001	30	(8.09 ± 0.02)E-5	44	(2.0 ± 0.1)E-6
16	0.004 ± 0.002	28	0.0014 ± 0.0008	40	(1.50 ± 0.04)E-4	65	(1.7 ± 0.7)E-4
17	0.005 ± 0.003	32	0.0028 ± 0.0006	50	(7.77 ± 0.04)E-4	70	(3.7 ± 0.4)E-4
18	0.006 ± 0.002	33	0.003 ± 0.002	55	(1.70 ± 0.06)E-3	80	(1.26 ± 0.08)E-3
19	0.0070 ± 0.0005	34	0.004 ± 0.001	70	(7.15 ± 0.03)E-3		
20	0.010 ± 0.006	35	0.006 ± 0.003				
21	0.012 ± 0.006	40	0.007 ± 0.001				
23	0.016 ± 0.006	50	0.014 ± 0.006				
24	0.016 ± 0.006	60	0.056 ± 0.009				
25	0.022 ± 0.006						
26	0.025 ± 0.004						
27	0.031 ± 0.004						
28	0.03 ± 0.01						
29	0.042 ± 0.01						
30	0.044 ± 0.01						
31	0.042 ± 0.01						
32	0.043 ± 0.01						
33	0.045 ± 0.007						

3. Sublimation rate constants calculated from TGA measurements.

SM TABLE II. Sublimation rate constants calculated from TGA measurements

TATP_1		2,4-DNT		TNT		RDX	
T (°C)	k (Kg*s^{-1})	T (°C)	k (Kg*s^{-1})	T (°C)	k (Kg*s^{-1})	T (°C)	k (Kg*s^{-1})
20	2.95E-11	25	5.06E-13	30	6.85E-14	55	9.43E-15
22	3.74E-11	27	6.42E-13	35	7.10E-14	60	1.21E-14
24	5.19E-11	29	1.00E-12	40	2.20E-13	65	2.43E-14
25	5.96E-11	30	1.30E-12	45	3.60E-13	75	6.00E-14
26	6.84E-11	35	1.70E-12	50	6.50E-13	80	1.40E-13
28	9.01E-11	40	3.30E-12	55	1.20E-12	85	2.90E-13
30	1.15E-10	45	6.10E-12	60	2.20E-12	90	3.20E-13
35	1.93E-10	50	1.10E-11	65	4.30E-12	95	5.00E-13
40	3.09E-10	55	1.90E-11	70	6.61E-12	100	6.90E-13
45	5.00E-10	60	3.20E-11	75	9.30E-12	105	1.42E-12
50	7.87E-10	65	5.10E-11	80	1.04E-11	110	1.54E-12
55	1.16E-09	70	8.27E-11	85	1.55E-11	115	2.31E-12
60	1.56E-09	75	1.05E-10	90	2.28E-11	120	2.79E-12
65	1.99E-09					125	4.20E-12

SM TABLE III. Sublimation rate constants calculated from TGA measurements

TATP_2		TATP_2		TATP_3		TATP_3		TATP_3	
T (°C)	k (Kg*s^{-1})	T (°C)	k (Kg*s^{-1})	T (°C)	k (Kg*s^{-1})	T (°C)	k (Kg*s^{-1})	T (°C)	k (Kg*s^{-1})
22	3.90E-11	54	1.21E-09	21.5	9.40E-11	35.0	5.62E-10	50.0	2.33E-09
23	4.23E-11	55	1.30E-09	22.0	1.01E-10	35.5	5.92E-10	50.5	2.44E-09
24	5.04E-11	56	1.40E-09	22.5	1.11E-10	36.0	6.23E-10	51.0	2.55E-09
25	5.98E-11	57	1.51E-09	23.0	1.21E-10	36.5	6.55E-10	51.5	2.66E-09
26	6.99E-11	58	1.61E-09	23.5	1.33E-10	37.0	6.92E-10	52.0	2.77E-09
27	8.12E-11	59	1.72E-09	23.0	1.19E-10	37.5	7.26E-10	52.5	2.89E-09
28	9.28E-11	60	1.82E-09	23.5	1.30E-10	38.0	7.61E-10	53.0	3.01E-09
29	1.05E-10	61	1.92E-09	24.0	1.41E-10	38.5	8.01E-10	53.5	3.12E-09
30	1.19E-10	62	2.01E-09	24.5	1.53E-10	39.0	8.40E-10	54.0	3.24E-09
31	1.35E-10	63	2.09E-09	25.0	1.64E-10	39.5	8.83E-10	54.5	3.37E-09
32	1.53E-10	64	2.14E-09	25.0	1.67E-10	40.0	9.26E-10	55.0	3.49E-09
33	1.73E-10			25.5	1.80E-10	40.0	9.85E-10	55.5	3.62E-09
34	1.94E-10			26.0	1.90E-10	40.5	1.02E-09	56.0	3.75E-09
35	2.16E-10			26.5	2.04E-10	41.0	1.06E-09	56.5	3.88E-09
36	2.42E-10			27.0	2.17E-10	41.5	1.11E-09	57.0	4.02E-09
37	2.70E-10			27.5	2.33E-10	42.0	1.15E-09	57.5	4.15E-09
38	3.00E-10			28.0	2.50E-10	42.5	1.20E-09	58.0	4.28E-09
39	3.31E-10			28.0	2.48E-10	43.0	1.25E-09	58.5	4.42E-09
40	3.65E-10			28.5	2.64E-10	43.5	1.31E-09	59.0	4.55E-09
41	4.22E-10			29.0	2.83E-10	44.0	1.37E-09	59.5	4.68E-09
42	4.65E-10			29.5	3.04E-10	44.5	1.43E-09	60.0	4.80E-09
43	5.09E-10			30.0	3.23E-10	45.0	1.49E-09	60.5	4.92E-09
44	5.46E-10			30.5	3.43E-10	45.5	1.56E-09	61.0	5.01E-09
45	5.87E-10			31.0	3.64E-10	46.0	1.63E-09	61.5	5.08E-09
46	6.40E-10			31.5	3.84E-10	46.5	1.71E-09	62.0	5.19E-09
47	6.98E-10			32.0	4.06E-10	47.0	1.79E-09	62.5	5.29E-09
48	7.58E-10			32.5	4.29E-10	47.5	1.87E-09	63.0	5.38E-09
49	8.21E-10			33.0	4.54E-10	48.0	1.95E-09	63.5	5.43E-09
50	8.89E-10			33.5	4.80E-10	48.5	2.04E-09	64.0	5.49E-09
51	9.58E-10			34.0	5.06E-10	49.0	2.13E-09	64.5	5.52E-09
52	1.04E-09			34.5	5.35E-10	49.5	2.23E-09	65.0	5.51E-09

4. Verification of the surface concentration of RDX by HPLC.

The total mass deposited onto the substrates was rinsed with acetonitrile, and the concentration was calculated from a High-Performance Liquid Chromatography (HPLC) calibration curve. The analysis was carried out using an Agilent C18 column (150 mm; 4.6 mm; 5 mm) and UV-Vis detector with wavelength set at 254 nm. A methanol/water (50/50 v/v) solvent mix was used as the mobile phase. The separation was run in the isocratic mode at 40 °C with a 1.0 mL/min flow rate and an injected volume of 10 mL.

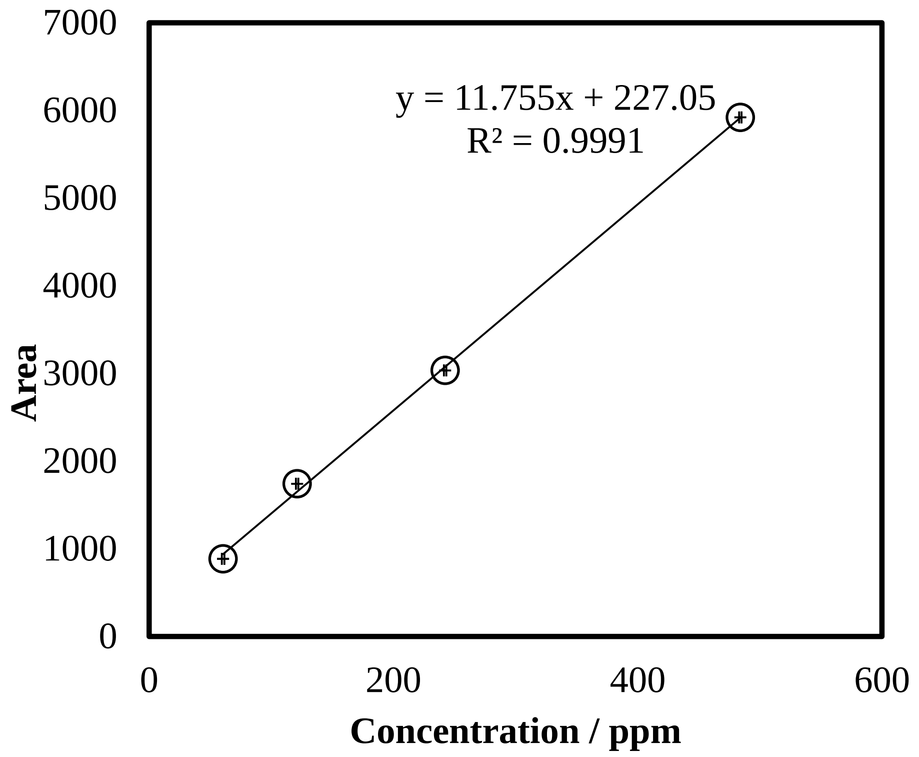

SM Fig. 8. Calibration curve for RDX by HPLC.

5. Kinetic and thermodynamic parameters for the various HEM studied.

SM TABLE IV. Kinetics parameters and thermodynamic functions calculated from GAP, TGA, and TPM

TATP

Exp	Temp. Range / T_{mean} (°C)	a J/mol	b J/mol·K	c J/mol·K	kJ/mol	R^2
GAP	14-33 / 20.9	27±2x10^{5}	8643±857	58±6x10^{3}	$\Delta_{sub}U$ 140±14	0.997
TGA_1	20-65 / 37.5	46±3x10^{4}	-1211±88	-82±6x10^{2}	$\Delta_{sub}H$ 83±5	0.999
TGA_2	22-64 / 44.0	45±1x10^{4}	-1500±36	-102±2x10^{2}	$\Delta_{sub}H$ 87±3	1.000
TGA_3	21-63 / 37.8	47±1x10^{4}	-1243±30	-85±2x10^{2}	$\Delta_{sub}H$ 86±2	1.000

2,4-DNT

Exp	Temp. Range / T_{mean} (°C)	Slope J/mol	Intercept J/mol·K	kJ/mol	R^2
GAP	23-35 / 36.0	(91±5)x10^{3}	-249±17	$\Delta_{sub}U$ 91±5	0.986
TGA	25-75 / 46.6	(94±2)x10^{3}	-114±4	$\Delta_{sub}H$ 94±2	0.998

TNT

Exp	Temp. Range/T_{mean} (°C)	Slope J/mol	Intercept J/mol·K	kJ/mol	R^2
GAP	40-70 / 55.0	(108±6)x10^{3}	-274±21	$\Delta_{sub}U$ 108±6	0.998
TGA	40-65 / 52.5	(95±3)x10^{3}	-92±10	$\Delta_{sub}H$ 95±3	0.997

RDX

Exp	Temp. Range/T_{mean} (°C)	Slope J/mol	Intercept J/mol·K	kJ/mol	R^2
GAP	22-80 / 56.2	(169±5)x10^{3}	-427±14	ΔU 169±5	0.998
TGA	55-125 / 90.0	(101±3)x10^{3}	-65±7	99±3	0.987

TPM	β_h °C/min	T_{Max} °C		$\Delta_{int}U$ kJ/mol
	5	117±2		
	10	142±3	$\Delta_{int}U$	19±1
	20	200±3		

6. GAP vs. GAO reflectance measurements for RDX.

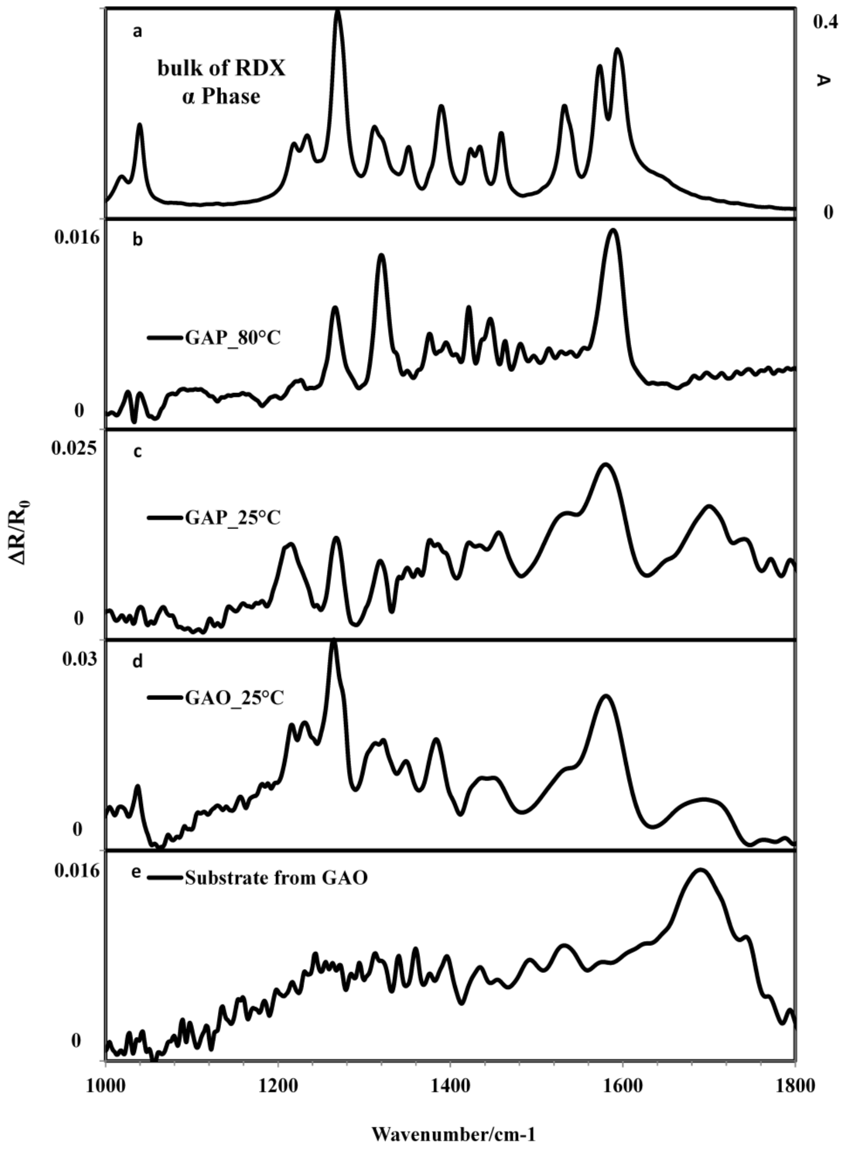

SM Fig. 9. (a) Plot of absorbance vs. wavenumber for RDX in KBr from macro-FTIR; (b) plot of ΔR/R vs. wavenumber of layers of RDX from GAP at 80 °C; (c) plot of ΔR/R vs. wavenumber of layers of RDX from GAP to 25°C; (d) plot of ΔR/R vs. wavenumber of layer of RDX from GAO to 25 °C; (e) Plot of ΔR/R vs. wavenumber of SS substrate by GAO.

Figs. 9b and 9c show the differences between the observed GAP spectra of RDX at 85 °C (GAP_85°C) and at 25 °C (GAP_25°C). The band at 1218 cm^{-1} (N-N stretching and ring stretching) has a higher intensity in the GAP_25°C and GAO_25°C than that of GAP_85°C (see Fig. 9d). A signal assigned to the substrate was observed at 1690 $cm^{-1,}$ and it is persistent in the GAP and GAO spectra at ambient temperature. This interfering signal can be due to a vibrational IR signal of the substrate. Significant differences between GAP and GAO spectra were not found on the range 1000 to 1600 cm^{-1}.

7. Comparison of signal-to-noise ratios for GAP and GAO measurements.

SM TABLE V. Signal to noise for GAP and GAO

	GAP		GAO	
Scan	noise	SNR	noise	SNR
1	0.00069	24	0.00160	4
5	0.00025	28	0.00130	5
10	0.00020	35	0.00120	7
20	0.00019	36	0.00035	20
50	0.00019	37	0.00035	23
120	0.00019	38	0.00030	23

8. Comparison of calculated $\Delta_{sub}H$ with literature values for TATP.

SM TABLE VI. Comparison of calculated Enthalpy of sublimation with literature values for TATP

	T_{mean} °C	Range °C	N of T/°C	ΔCp kJ/mol·K	$\Delta_{sub}H$ kJ/mol	Prediction with models to T_{mean}		
						$\Delta_{sub}H$ TGA_1	$\Delta_{sub}H$ TGA_2	$\Delta_{sub}H$ TGA_3
	25.0					**99 ± 6**	**107 ± 2**	**102 ± 2**
GAP	20.9	14- 33	19	-8.6 ± 0.9	144 ± 14	-	-	-
TGA_1	37.5	20- 65	14	-1.21 ± 0.09	83 ± 5	-	-	-
TGA_2	44.0	22- 64	42	-1.50 ± 0.04	87 ± 2	-	-	-
TGA_3	37.8	21- 63	88	-1.24 ± 0.03	85 ± 2	-	-	-
Damour et al 2010	14.3	-3- 34	27	0.3 ± 0.1	86.2 ± 1	111 ± 8*	122 ± 3*	114 ± 3*
Ramirez et al 2006	50	25- 75	7	-0.75 ± 0.08	85.8	72 ± 5	74 ± 2	74 ± 2
Felix et al. 2011	50	25-75	8	-	72.1	72 ± 5	74 ± 2	74 ± 2
Oxley et al. 2005	40.0	12- 58	6	1.5 ± 0.9	109	80 ± 5	84 ± 1	83 ± 2
Oxley et al. 2009	32.2	15- 50	7	0.5 ± 0.6	73	90 ± 6	96 ± 2	93 ± 2
Dunayevskiy et al. 2007	0.0	-30- 30	-	-	81.3	129 ± 10*	144 ± 4*	133 ± 4*
Espinosa-Fuentes et al. 2015	46	22-70	32	1.5	103.8 ± 0.2	75 ± 5	78 ± 2	78 ± 2

* found by extrapolation

9. Equations

$$k = k^0 \exp\left(-\frac{\Delta E}{R_g}\frac{1}{T}\right)$$

$$lnk = lnk^0 - \frac{\Delta E}{R_g}\frac{1}{T}$$

$$\frac{\partial Lnk}{\partial\left(\frac{1}{T}\right)} = -\frac{\Delta E}{R_g}$$

$$dH = dU + d(pV) = dU + pdV + Vdp \approx \Delta H = \Delta U + p\Delta V$$

$$\Delta V = V_{gas} - V_{solid} \approx V_{gas}$$

$$\Delta H = \Delta U + pV_{gas} = \Delta U + R_g T$$

$$\Delta H = -R_g\left(\frac{\partial Lnk}{\partial\left(\frac{1}{T}\right)} + T\right)$$

10. Calculation of Uncertainties of the enthalpy in the media temperature

Uncertainties (σ) in ΔH were calculated,

$$\sigma_y^2 = \sigma_{yI}^2 + \sigma_{yD}^2$$

$$\sigma_y^2 = (\delta_T b)^2 + [(T - T_{mean})s_b]^2 + \left(\frac{s_r}{-R_g ln(\zeta)}\Delta H\right)^2 = \sigma_{\Delta H}^2$$

where σ_{yD} is a direct contribution from the model and σ_{yI} is an indirect contribution calculated from the propagation of uncertainties.δ_T is of the order of 0.001 K/T for TGA and 0.1 K/T for GAP, S_b is the standard deviation of b, σ_{yD} is ΔH_{mean} plus the standard error of the model divided by $-R_g ln(\zeta)$, where ζ is k or ν_s. σ at media temperatures ($\sigma\Delta H_{mean}$) can be obtained using:

$$\sigma_{\Delta H}^2 = (\delta_T b)^2 + \left(\frac{s_r}{-R_g ln(\zeta_{mean})}\Delta H_{mean}\right)^2$$

11.Size of droplets of metastable phase for TNT and 2,4 DNT

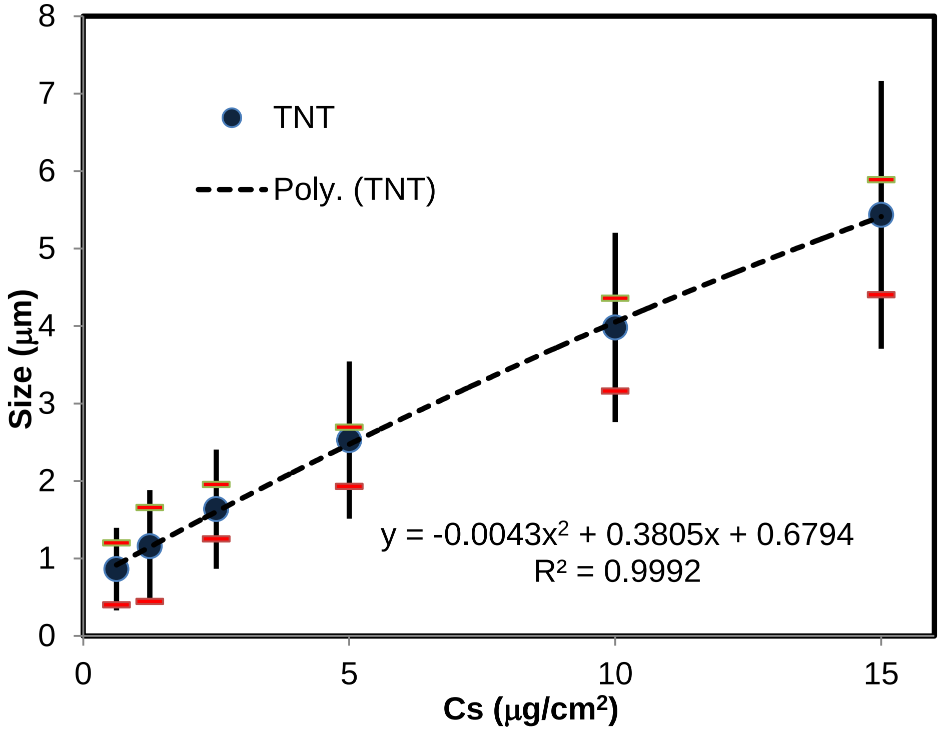

SM Fig. 10. The plot of droplet size vs. surface concentration for TNT. The figure shows the standard deviation, (black line), first and third quartile (orange), and quadratic fit (dotted line).

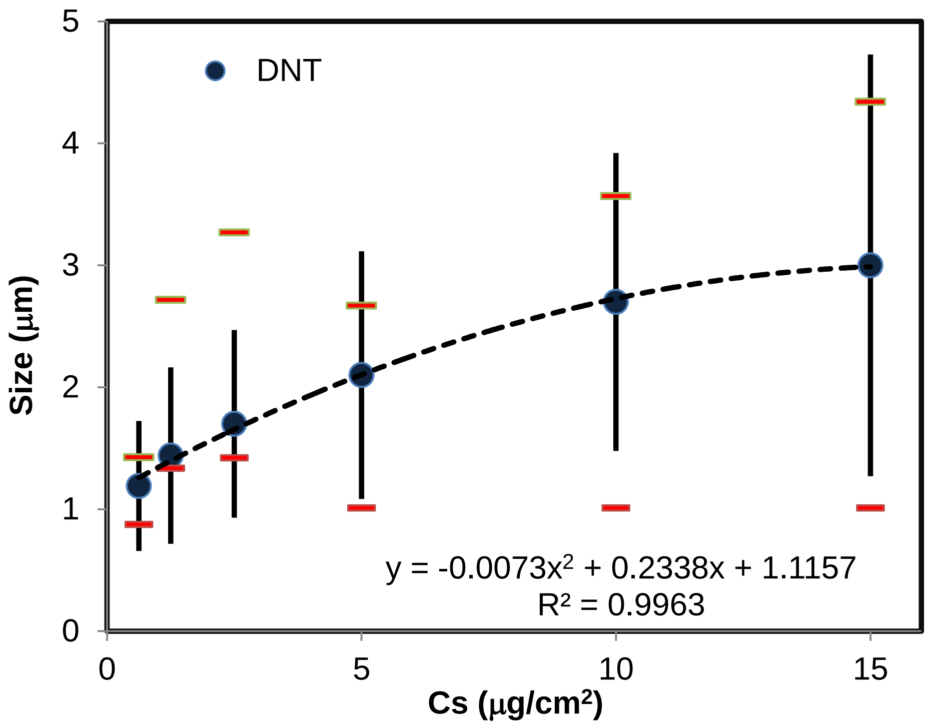

SM Fig. 11. The plot of droplet size vs. surface concentration for 2,4 DNT. The figure shows the standard deviation, (black), first and third quartile (orange) and quadratic fit (dotted line).

12. Sublimation experiment for benzoic acid by TGA instrument

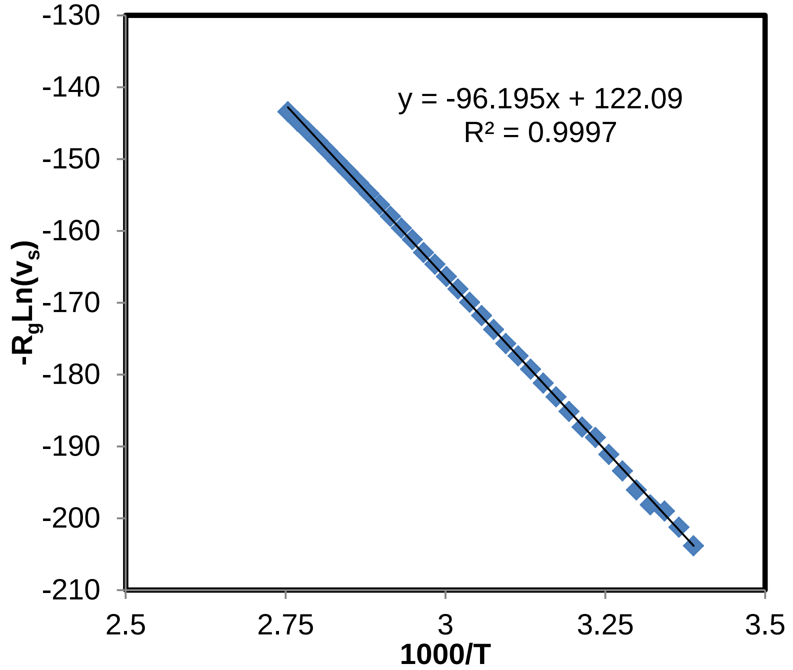

SM Fig. 12. Arrhenius plots of TGA data used to obtain the sublimation rates for Benzoic acid.
